# Quasi-Static Modeling Framework for Soft Bellow-Based Biomimetic Actuators

**DOI:** 10.3390/biomimetics9030160

**Published:** 2024-03-04

**Authors:** Kelvin HoLam Heung, Ting Lei, Kaixin Liang, Jiye Xu, Joonoh Seo, Heng Li

**Affiliations:** Department of Building and Real Estate, Hong Kong Polytechnic University, Hong Kong; tinglei@polyu.edu.hk (T.L.); karenleung@163.com (K.L.); xujiye2021@163.com (J.X.); joonoh.seo@polyu.edu.hk (J.S.); heng.li@polyu.edu.hk (H.L.)

**Keywords:** soft robots, finite element method (FEM), pneumatic extension actuators, elongation, analytical modeling

## Abstract

Soft robots that incorporate elastomeric matrices and flexible materials have gained attention for their unique capabilities, surpassing those of rigid robots, with increased degrees of freedom and movement. Research has highlighted the adaptability, agility, and sensitivity of soft robotic actuators in various applications, including industrial grippers, locomotive robots, wearable assistive devices, and more. It has been demonstrated that bellow-shaped actuators exhibit greater efficiency compared to uniformly shaped fiber-reinforced actuators as they require less input pressure to achieve a comparable range of motion (ROM). Nevertheless, the mathematical quantification of the performance of bellow-based soft fluidic actuators is not well established due to their inherent non-uniform and complex structure, particularly when compared to fiber-reinforced actuators. Furthermore, the design of bellow dimensions is mostly based on intuition without standardized guidance and criteria. This article presents a comprehensive description of the quasi-static analytical modeling process used to analyze bellow-based soft actuators with linear extension. The results of the models are validated through finite element method (FEM) simulations and experimental testing, considering elongation in free space under fluidic pressurization. This study facilitates the determination of optimal geometrical parameters for bellow-based actuators, allowing for effective biomimetic robot design optimization and performance prediction.

## 1. Introduction

Soft robotics, as a subfield of robotics, focuses on the advancement and utilization of robots constructed from pliable and flexible materials like silicone, rubber, and plastics [1,2]. These robots diverge from conventional mechanical counterparts that primarily rely on rigid materials, resulting in limited flexibility. Instead, soft robots are specifically engineered to emulate the movements and functionalities observed in a diverse range of natural organisms, ranging from octopuses and worms to human muscles [3]. Soft robotic actuators possess numerous advantages over traditional mechanical electric motors and rigid hydraulic actuators. These advantages include increased compliance, lower inherent stiffness, lighter weight, lower cost, and the ability to provide customizable motion [4,5,6,7,8]. Pioneering researchers such as Shi et al. [9], Heung et al. [10], and Keplinger et al. [11] have contributed to the development of soft actuators. Their work has involved the utilization of various materials, including electroactive polymers, shape memory alloys, and elastomers, to engineer soft actuators capable of specific motions such as bending, elongation, twisting, and more. These motions are achieved by employing different actuation stimuli, such as electrical charges, chemical reactions, or pressurized fluids [12]. Among the diverse range of flexible actuators, fluidic-driven elastomeric actuators have gained considerable interest. This is primarily due to their superior power-to-weight ratio and the convenience of fabrication through traditional molding and casting processes using silicone rubber [13,14]. By applying pressure to the embedded chambers within soft actuators, these fluidic-driven systems can deform towards directions associated with low stiffness, enabling motions including bending, extending, or twisting.

Developing a motion prediction model for soft actuators is crucial to guide efficient design processes [15]. Earlier investigations focused on modeling the behavior of PneuNets and fiber-reinforced soft actuators under pressurization [16,17], specifically examining the correlation between angle–pressure and force–pressure relationships. However, the deformable nature of these systems presents a challenge as soft bodies can have infinite degrees of freedom (DOF), limiting the effectiveness of analytical methods. The analytical modeling process for fiber-reinforced actuators is regarded as simpler due to their straightforward and uniform tubular structure, as opposed to the more complex bellow-shaped fluidic soft actuators. Comparatively, the intricate structure of bellow-shaped soft fluidic actuators poses challenges in developing mathematical equations that predict their output performance. Empirical approaches based on physical experiments and the finite element method (FEM) were utilized first, and specifically, a recurrent neural network-based adaptive unscented Kalman filter architecture was proposed to estimate the kinematics in a soft bending actuator [18]. This scheme eliminated the need for complex model deduction but may suffer from inconsistency and the requirement of additional training data, which can be costly and time-consuming. Recent efforts made by Felt et al. and Ma et al. have employed virtual work principles and thin-plate bending theory, respectively, to establish robust quasi-static analytical models for bellow actuators in relation to input pressure [19,20]. However, to simplify the development of analytical equations, the assumption of material linearity was made regarding the elastomers used. Nevertheless, the response of adopted elastomers tends to be nonlinear as the strain applied to the actuator structure during motion increases [21]. This nonlinearity can result in divergent outcomes when bellow-shaped soft fluidic actuators undergo significant deformation with an increasing amount of input pressure. Despite ongoing efforts, there have been limited advancements in the development of various mathematical methods that can comprehensively elucidate and predict the deformations exhibited by soft bellow-type pneumatic actuators.

This article presents the design of bellow-shaped soft actuators made of elastomers, which are specifically programmed to undergo elongation when subjected to fluidic pressurization. The crest of the bellow structure incorporates rigid constraints that restrict the expansion of the entire cavity while enabling the root of the bellow actuator to expand. This configuration facilitates overall actuation extension when pressurized fluid is applied to the cavities. By considering the expansion of multiple roots along the bellow actuator as individual expanding cavities with infinitesimally small widths, a quasi-static mathematical model is developed to characterize the elongation displacement of the soft bellow actuator in relation to the input pressure applied to the actuation cavity. Experimental results are presented, including elongation displacement and output force measurements, as well as validation through FEM and analytical results for accuracy validation. In comparison to tubular actuators, e.g., fiber-reinforced actuators, that possess a simpler mechanical structure, bellow-shaped soft actuators offer distinct advantages: they experience minimal strain and volume changes during actuation, resulting in reduced fatigue and improved durability; they enable faster actuation; and they can achieve the same angle of motion at lower pressures [22,23,24].

The structure of the remainder of this paper is as follows. Section 2 outlines the design and fabrication of the bellow-shaped soft extending actuators. Section 3 introduces the analytical modeling approach employed for the actuators. Section 4 presents the results of the model analysis, highlighting its significance. Finally, Section 5 concludes the research and provides a glimpse into potential future developments of bellow-shaped actuators across various applications.

## 2. Soft Bellow-Shaped Actuator

**Figure 1:**
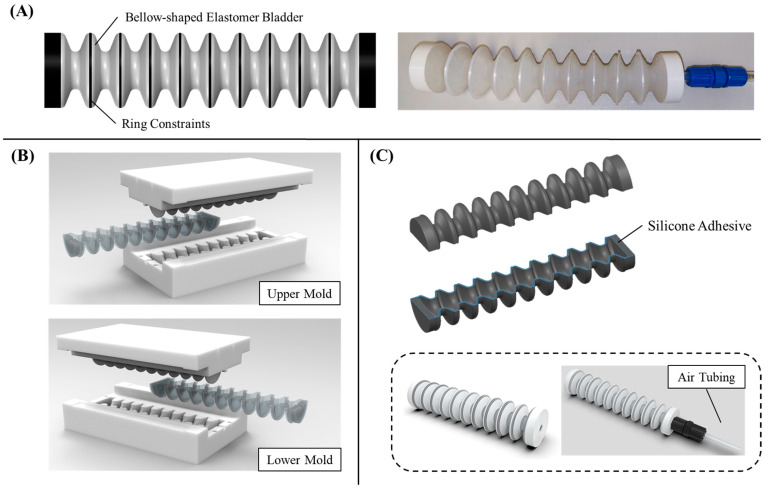
(**A**) General conceptual and real prototype design of the bellow-shaped elongation soft actuator with multiple rigid ring constraints embedded around the crests, (**B**) cross-section area view showing the fabrication of the actuator, and (**C**) the assembly of the soft elongation actuator using silicone adhesive.

***Actuator Design.*** Our soft bellow actuators retain the advantages of lightweight and lower inherent impedance compared to electric counterparts (see Figure 1A). These actuators consist of an internal cavity with undulations, comprising multiple crests and roots for the application of pressurized fluid. When the cavity is pressurized, the actuator extends in the direction of its length. This allows pneumatic sources to effectively control the extension of the actuators, resulting in a larger elongation displacement compared to traditional fiber-reinforced actuators. To ensure uniform expansion of the cavities, we employ rigid ring constraints around the crests, preventing any irregular expansion. However, the roots of the bellow remain unconstrained to enable unimpeded expansion during the unfolding of the actuator for extension. Furthermore, we incorporate anchors in the crests to securely attach the ring constraints to the surface of the actuator without detachment upon multiple actuation overtime.

***Fabrication.*** The bellow-shaped soft actuator is manufactured using the virtual lost wax casting method [25] as the actuator body incorporates multiple tapered edges around its crests. In contrast to traditional lost wax casting, this process utilizes a 3D-printed mold instead of wax. The detailed fabrication process for these soft actuators can be found in [26]. It involves printing the mold, filling it with a silicone rubber mixture, curing the rubber, and demolding the actuator (see Figure 1B). The actuator is made from pliable silicone rubber, specifically Dragon Skin 10 and 30 (Smooth-On, Inc., Macungie, PA, USA). These materials offer significant elongation capabilities (up to 1000% and 364%, respectively) and hardness (Shore A 10 and 30), which enable the actuator to support large deformations while preventing rupture in its bent state [10,27]. For multiple ring constraints located at the crests, they are 3D-printed using polylactide (PLA).

To simplify the demolding process of the actuators from the mold cores, the bellow actuator is divided into two identical halves along the radial plane (see Figure 1C). Each half is fabricated and demolded separately, and then they are joined together along the wall of the actuator using silicone adhesive (Sil-Poxy, Smooth-On, Inc., Macungie, PA, USA). This approach ensures the easy and efficient assembly of the actuator components. Eventually, the actuator has an approximate weight of 19 g.

## 3. Mathematical Modeling for Elongation Estimation

**Figure 2:**
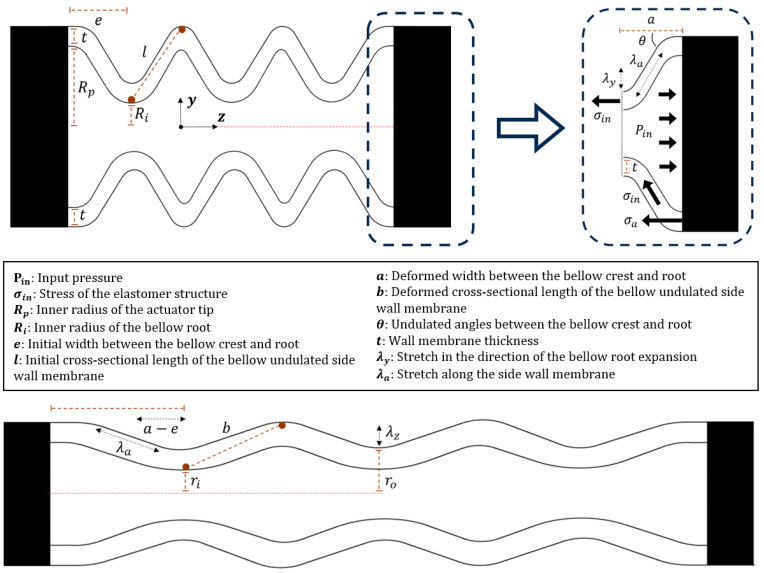
Definition of the bellow dimensions before and after deformation upon fluid pressurization.

A mathematical model has been developed and presented which illustrates the correlation between the input pressure and elongation displacement of the actuator. The model considers the force equilibrium between the extension force created by the input pressure and the resistive force that arises from the elasticity of the elastomer body (see Figure 2).

***Extension force created from fluid injection*.** The actuator undergoes elongation when pressure is applied, and the extent of elongation is influenced by the pressure level within the undulated cavity. It is assumed that the tip of the actuator does not experience any deformation across its cross-section when subjected to pressurized fluid [28,29]. The resulting force causing the actuator to extend can be determined by considering the pressure applied to the actuator.

***(1)***  $F_{pressure}$**, extension force for actuator elongation.**

(1) $$F_{Pressure}=P_{in}\cdot \pi \cdot {R_{p}}^{2}$$

where $P_{in}$ is the input pressure to the cavity, and $R_{p}$ represents the internal area of the actuator subjected to pressure $P_{in}$.

***Resistive force created by the elastomer body*.** The elongation of the actuator leads to the elastomer body generating resistance in the opposite direction to the extension. The resulting force caused by the resistance opposing the extension of the soft actuator is given by

***(2)***  $F_{resistive}$**, resistive force opposing actuator elongation.**

As we adopted both Dragon Skin 10 and 30 for the fabrication of the actuators, their stress–stretch characteristic can be described using the Yeoh third-order (Dragon Skin 10) and Ogden first-order (Dragon Skin 30) hyper-elastic models, respectively [30]. The strain energy is expressed as

*Dragon Skin 10 (Yeoh third-order model):*

(2) $$W_{10}=\sum_{i=1}^{3}C_{i}\cdot {\left(I_{1}-3\right)}^{i}$$

where $C_{i}$ denotes the material constants to be determined by fitting the stress–strain data of the adopted silicone rubber into the strain energy function $W_{10}$, and $I_{1}$ is the strain invariant of silicone rubber.

*Dragon Skin 30 (Ogden first-order model):*

(3) $$W_{30}=\frac{2\mu }{{\alpha_{1}}^{2}}\left({\lambda_{1}}^{\alpha_{1}}+{\lambda_{2}}^{\alpha_{1}}+{\lambda_{3}}^{\alpha_{1}}-3\right)$$

where the material coefficient $\alpha_{1}$ is the strain-hardening exponent, and μ is the small strain shear modulus. The calculation of the elongation displacement of the bellow actuator involves considering the combined effect of two key factors, which are (a) the expansion of the bellow roots, which causes the unfolding of the undulated bellow cavity, and (b) the lengthened undulated bellow side wall membranes resulting from the stretching of the elastomeric materials when subjected to pressurized fluid.

**(a)**  **Expansion of bellow roots**

The expanding sections, i.e., multiple roots, of the bellow actuator can be represented as cylindrical rubber tubes with differential widths that are subjected to internal input pressure. The principal stretches, denoted as $\lambda_{r}$, $\lambda_{\theta }$, and $\lambda_{z}$, represent the elongation ratios experienced by the cylindrical rubber tube in the radial, circumferential, and axial directions, respectively, due to the internal pressure. Assume that neglectable elongation occurs at the roots; then, the stretches are given by [31]

(4) $$\lambda_{r}=\frac{1}{\lambda }\;\lambda_{\theta }=\lambda \;\lambda_{z}=1$$

where $\lambda_{r}\lambda_{\theta }\lambda_{z}$ = 1 considering the incompressibility of general silicone rubber. In the expanded configuration of the expanding sections, the radial stress $\sigma_{r}$ is zero at the outer surface of the membrane and equal to the internal input pressure at the inner surface. Additionally, in the absence of body forces within the expanding sections, the equilibrium condition between radial stress $\sigma_{r}$ and circumferential stress $\sigma_{\theta }$ can be described by the equation specified in reference [31].

(5) $$\frac{d\sigma_{r}}{dr}=\frac{1}{r}\left({\sigma_{\theta }-\sigma }_{r}\right)$$

Therefore, the relationship between the input pressure and expansion of multiple roots can be obtained by solving

(6) $$\begin{array}{cc} P_{in} & =-\int_{-P}^{0}d\sigma_{r}\\ & =-\int_{r_{i}}^{r_{o}}\frac{1}{r}\left({\sigma_{\theta }-\sigma }_{r}\right)dr \end{array}$$

For the general incompressible hyper-elastic material model of silicone rubber, the Cauchy stress throughout the wall membrane can be expressed as

(7) $$\sigma_{r}=-p+\frac{2}{\lambda^{2}}\frac{\partial W}{\partial I_{1}}\;\sigma_{\theta }=-p+2\lambda^{2}\frac{\partial W}{\partial I_{1}}$$

Depending on the adopted material models, the derivative of the strain energy function can be represented as

*Dragon Skin 10 (Yeoh third-order model):*

(8) $$\frac{\partial W_{10}}{\partial I_{1}}=\sum_{i=1}^{3}{i\cdot C}_{i}{\left(\lambda^{-2}+\lambda^{2}-2\right)}^{i-1}$$

*Dragon Skin 30 (Ogden first-order model):*

(9) $$\frac{\partial W_{30}}{\partial I_{1}}=\frac{\mu_{1}{\lambda_{j}}^{\alpha_{1}}}{\lambda_{j}}$$

that

(10) j=r,θ,z

and p is the Lagrange multiplier determined by considering the internal body forces within the elastomer structure and can be ignored for the calculation of the relationship between the input pressure and the expansion of the roots.

Therefore, by following the same previous steps to solve the equilibrium condition in Equations (5)–(7) we obtain

*Dragon Skin 10 (Yeoh third-order model):*

(11) $$\begin{array}{cc} P_{in} & =-\int_{r_{i}}^{r_{o}}\frac{2}{r}\frac{\partial W_{10}}{\partial I_{1}}\cdot \left(\lambda^{2}-\lambda^{-2}\right)dr\\ & =-\int_{\lambda_{r_{i}}}^{\lambda_{r_{o}}}\frac{2}{\lambda }\frac{\partial W_{10}}{\partial I_{1}}\cdot \left(\lambda^{2}-\lambda^{-2}\right)\cdot \frac{1}{1-\lambda^{2}}d\lambda \end{array}$$

*Dragon Skin 30 (Ogden first-order model):*

(12) $$\begin{array}{cc} P_{in} & =-\int_{r_{i}}^{r_{o}}\frac{2}{r}\frac{\partial W_{30}}{\partial I_{1}}\cdot \left(\lambda^{2}-\lambda^{-2}\right)dr\\ & =-\int_{\lambda_{r_{i}}}^{\lambda_{r_{o}}}\frac{2}{\lambda }\frac{\partial W_{30}}{\partial I_{1}}\cdot \left(\lambda^{2}-\lambda^{-2}\right)\cdot \frac{1}{1-\lambda^{2}}d\lambda \end{array}$$

which

(13) $$\begin{array}{ccc} dr & = & \frac{R_{i}}{1-\lambda^{2}}d\lambda \\ \lambda_{r_{i}} & = & \frac{r_{i}}{R_{i}}\\ \lambda_{r_{o}} & = & \frac{r_{o}}{R_{i}+t} \end{array}$$

Again, $R_{i}$ and $R_{i}+t$ are the internal and external radii of the bellow roots before expansion, and $r_{i}$ and $r_{o}$ are the internal and external radii after expansion of the roots. To further present $\lambda_{r_{i}}$ and $\lambda_{r_{o}}$ in terms of λ, they can be rewritten as [31]

(14) $$\begin{array}{ccc} \lambda_{r_{o}} & = & \lambda \\ \lambda_{r_{i}} & = & \lambda_{r_{o}}+\frac{\left(2\epsilon +\epsilon^{2}\right)\cdot \left({\lambda_{r_{o}}}^{2}-1\right)}{2\lambda_{r_{o}}} \end{array}$$

which

(15) $$\epsilon =\frac{t}{R_{i}}$$

As mentioned, the length of the bellow roots is assumed to be constant during the expansion; the relationship between the internal input pressure $P_{in}$ and circumferential stretch $\left(\lambda =\lambda_{y}\right)$ representing the expansion of the roots can be simplified into

*Dragon Skin 10 (Yeoh third-order model):*

(16) $$P_{in}=-\int_{\lambda +\frac{\left(2\epsilon +\epsilon^{2}\right)\cdot \left({\lambda_{r_{o}}}^{2}-1\right)}{2\lambda_{r_{o}}}}^{\lambda }\frac{2}{\lambda }\frac{\partial W_{10}}{\partial I_{1}}\cdot (\lambda^{2}-\frac{1}{\lambda^{2}})\cdot \frac{1}{1-\lambda^{2}}d\lambda$$

*Dragon Skin 30 (Ogden first-order model):*

(17) $$P_{in}=-\int_{\lambda +\frac{\left(2\epsilon +\epsilon^{2}\right)\cdot \left({\lambda_{r_{o}}}^{2}-1\right)}{2\lambda_{r_{o}}}}^{\lambda }\frac{2}{\lambda }\frac{\partial W_{30}}{\partial I_{1}}\cdot (\lambda^{2}-\frac{1}{\lambda^{2}})\cdot \frac{1}{1-\lambda^{2}}d\lambda$$

Since Equations (16) and (17) are complex, the integrals are solved empirically by gradually increasing the circumferential stretch $\lambda_{y}$ from λ=1, with an increment of 0.1 for each iteration until full expansion of the bellow roots occurs. The calculated $P_{in}$ with respect to the corresponding circumferential stretch is further recorded for the analytical relationship between the input pressure and deformation of the bellow actuator. Reference [17] provides further details on the numerical approach used for solving this mathematical relationship.

**(b)**  **Lengthened undulated bellow side wall membranes**

When the bellow actuator is actuated with pressurized fluid in the cavity, the undulated side wall membranes experience a substantial elongation. This elongation is a result of the pressure-induced deformation of the membranes, causing them to stretch and extend. In this case, the circumferential strain $\lambda_{\theta }$ becomes negligible due to the wrapping of ring constraints. Therefore, the principal stretches $\lambda_{r}$, $\lambda_{\theta }$, and $\lambda_{z}$ become [27]

(18) $$\lambda_{r}=\frac{1}{\lambda }\;\lambda_{\theta }=1\;\lambda_{z}=\lambda$$

The axial stretch ($\lambda =\lambda_{a}$) that contributes to the elongation of the bellow side wall membranes is given by

*Stress of Dragon Skin 10 (Yeoh third-order model):*

(19) $$\begin{array}{cc} \sigma_{a10} & =\sigma_{in10}\cdot \cos\theta \\ & =2(\lambda^{2}-\frac{1}{\lambda^{2}})\frac{\partial W_{10}}{\partial I_{1}}\cdot \frac{a}{b} \end{array}$$

*Stress of Dragon Skin 30 (Ogden first-order model):*

(20) $$\sigma_{a30}=\mu_{1}\left(\lambda^{\alpha_{1}}-\lambda^{{-\alpha }_{1}}\right)\cdot \frac{a}{b}$$

where

(21) $$\begin{array}{c} a=\sqrt{b^{2}-{\left(R_{p}-{\lambda_{y}R}_{i}-t\right)}^{2}}\\ b=\lambda_{a}\cdot l \end{array}$$

Again, $\lambda_{y}$ denotes the strain in the direction of the expansion of the bellow roots from section (a), and $\lambda_{a}$ denotes the strain along the direction of the bellow wall membrane axially.

Eventually, the axial elongation $\lambda_{x}$ can be determined by considering $F_{resistive}=F_{pressure}$, which further discloses

(22) $$\frac{\lambda_{a}\cdot l{\cdot \sigma }_{a}}{\sqrt{{\left(\lambda_{a}\cdot l\right)}^{2}-{\left(R_{p}-{\lambda_{y}R}_{i}-t\right)}^{2}}}=\frac{P_{in}\cdot {R_{p}}^{2}}{\left[{\left(R_{i}+t\right)}^{2}-{R_{i}}^{2}\right]}$$

and the overall elongation displacement Δx of the soft actuator resulting from the expansion of the bellow roots for each bellow pitch can be determined by

(23) $$\Delta x=2n\left\{\sqrt{{{\left[\lambda_{a}\cdot l\right]}^{2}-\left[{(R}_{p}+t)-\lambda_{y}(R_{i}+t)\right]}^{2}}-e\right\}$$

**Figure 3:**
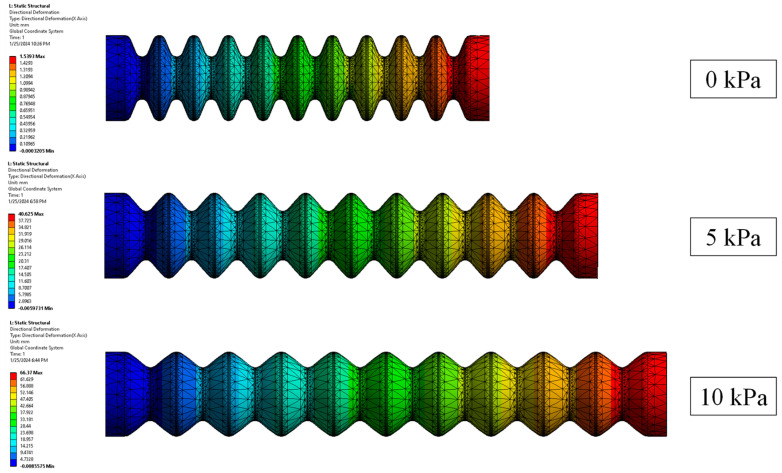
Flowchart presenting the overall setup of the FEM simulation.

**Figure 4:**
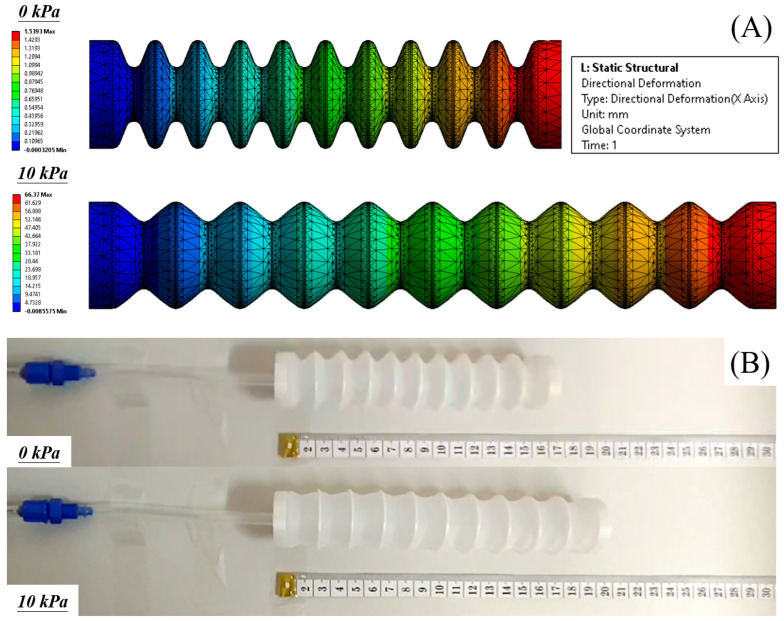
(**A**) FEM-simulated extension of an example soft bellow actuator and (**B**) example actuator elongation upon fluid pressurization at pressure input of 0 and 10 kPa.

***Finite Element Method (FEM) Modeling.*** We utilize ANSYS Workbench 15 to establish a 3D FEM model for the bellow actuator (see Figure 3). The model is subjected to a static structural analysis to determine the elongation displacement under various input pressures. The settings of the model were reported in our previous work [10,27]. To begin with, the procedure entails assigning appropriate materials to the elastomer body of the bellow actuator and the rigid ring constraints. An Yeoh third-order hyper-elastic model (N = 3) with coefficients of $C_{1}$ = 36,000 Pa, $C_{2}$ = 2500 Pa, and $C_{3}$ = 230 Pa is used to model Dragon Skin 10. Furthermore, an Ogden first-order hyper-elastic model with coefficients of $\mu_{1}$ = 75,449 Pa and $\alpha_{1}$ = 5.836 is used to model Dragon Skin 30 [27,30]. For the ring constraints (assumed as polyethylene), the material properties are directly obtained from ANSYS Engineering Data Sources. Then, it further involves establishing several boundary conditions that accurately simulate real-world scenarios of the actuator during elongation. In the settings, the proximal end of the bellow actuator is subjected to a fixed support condition. Bonded contact is established between the ring constraints and the elastomer actuator body. To enhance computational efficiency, the inlet channels for supplying fluid into the cavity are disregarded, and pressure is directly applied to the walls of the air chambers. Eventually, deformation results pertaining to horizontal elongation are obtained. To ensure accurate results while obtaining the deformation displacement of the bellow actuator (see Figure 4A), 3D 10-Node tetrahedral structural solid elements (ANSYS element type SOLID187) are used for both the elastomeric actuator body and the ring constraints.

## 4. Experimental Evaluation

**Figure 5:**
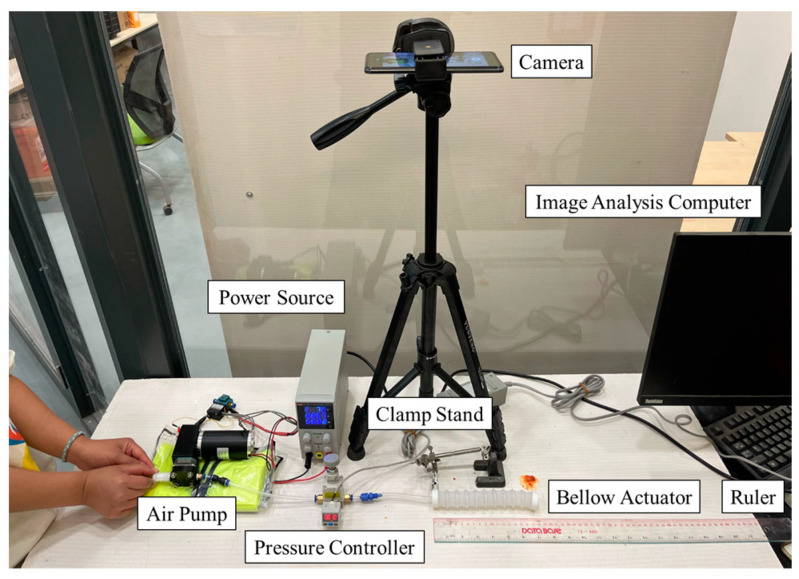
Experimental setup for measuring the elongation of the bellow actuator prototypes.

***Measurement Setup.*** An experimental platform is developed with a pneumatic control setup and camera system to measure the free space deformation when subjected to fluid pressurization (see Figure 5), as previously reported in our research [9,10,15,27]. Within the platform, the proximal cap with the air inlet of the bellow-based soft actuator is clamped in a rigid fixture, emulating the boundary constraints defined in the FEM simulations. The distal cap of the actuator is free to extend towards its horizontal direction. It is important to note that the actuator is placed horizontally on a flat surface instead of in free space to prevent deflection caused by gravitational forces, which could affect the accuracy of the measured elongation results. A high-definition camera is used to monitor the actuator from above, allowing for the recording of the extension trajectory of the actuator tip. A metric ruler is also placed on the flat surface with the actuator, enabling direct observation of the actual elongation displacement. The camera captures the movement of the actuator, and the recorded images are analyzed using image analysis software (ImageJ 1.54d, National Institute of Health, Bethesda, MD, USA) on a computer. The bellow actuator is further gradually supplied with air pressure through an air pump (BTC Diaphragm Pump, Parker Hannifin Corporation, Mayfield Heights, OH, USA). The air pressure is regulated using a pressure meter (ZSE20C(F), SMC Pneumatic, Tokyo, Japan). Moreover, the setup incorporates a pressure regulator (IR2020-02BG, SMC Pneumatic, Tokyo, Japan), which allows for manual adjustment of the air pressure supplied to the actuator (see Figure 5). The pressure value is displayed on the screen of the pressure sensor. To facilitate operation, a 12V voltage source is connected to the experimental platform.

**Figure 6:**
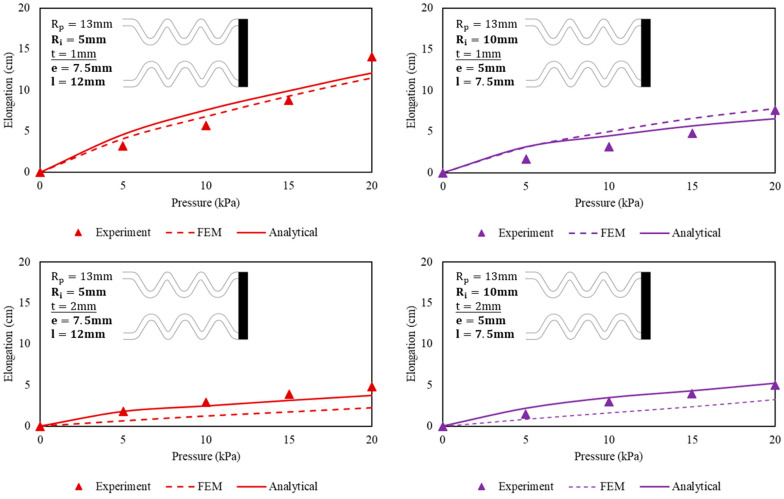
Pressure-elongation relationship of the undulated soft bellow-based actuator corresponding to four different dimensions.

In the experiments, four sets of actuators are evaluated (see Figure 6). They are tested for elongation displacement in free space. During the measurement, the proximal end of the actuator that is connected with the air tube is clamped. The elongation displacement of the actuator is captured and measured using image analysis software.

***Elongation Displacement in Free Space.*** During the measurement process, the undulated cavity within the bellow actuator is exposed to a range of pressures spanning from 0 kPa to 20 kPa, with increments of 5 kPa. The resulting elongation displacements are then compared with the predictions made by both analytical models and FEM simulations. To minimize the influence of gravity-induced deflection, the actuators are placed horizontally on a table during the experiments. The maximum allowable input pressure for the actuator is determined by the point at which the bellow roots are fully expanded, resulting in the actuator reaching its maximum elongation. It is important to note that any results obtained after the actuators have reached full elongation are considered irrelevant and will be disregarded. At this point, the actuators have lost their ability to further extend as they are completely unfolded. It is worth mentioning that primary deformation occurs in the bulging regions near the root rather than in the extension of the actuator itself.

***FEM Simulations.*** Both FEM simulations and analytical models exhibited an increasing trend of elongation with increasing input pressure, as well as validating the relationship between elongation displacement and actuator wall membrane thickness at a given input pressure level. When subjected to a pressure of 20 kPa, the actuator reached its maximum elongation during FEM simulations. Further increasing the input pressure in the FEM setup resulted in a failure to converge the solution, possibly due to an excessive force imbalance in the model caused by the potential bulging of multiple roots. Specifically, the 1 mm actuators demonstrated elongations of 11.5 cm and 7.8 cm (analytical results of 12.1 cm and 6.6 cm) when subjected to 20 kPa pressure. In comparison, the 2 mm actuators displayed lesser elongations: 2.3 cm and 3.3 cm (analytical results of 3.8 cm and 5.3 cm) under the same 20 kPa pressure level. The largest difference of 2.6 cm between the FEM and experimental results was observed for the 1 mm actuator with an inner radius of 5 mm, pressurized to 20 kPa. In this investigation, 20 kPa was thereby chosen as the maximum allowable input pressure for all the bellow actuator samples hereafter as larger input pressure resulted in difficulties in solution convergence. Also, further increasing the air pressure significantly contributed to complete extension of the actuator, rendering any results obtained thereafter irrelevant due to the loss of unfolding function. Moreover, excessive input pressure mainly caused irregular bulging effects.

***Experimental Results.*** Both experimental findings and analytical models also exhibited an increasing trend of elongation with increasing input pressure. Under the preset maximum input pressure of 20 kPa, the actuator experienced negligible bulging during extension. Instead, the predominant deformation occurred during the unfolding of the bellow actuator as the roots expanded within 0 to 20 kPa. The 1 mm actuator prototypes demonstrated elongations of 14.1 cm and 7.6 cm (analytical results of 12.1 cm and 6.6 cm) when subjected to 20 kPa pressure. In comparison, the 2 mm actuator prototypes also displayed lesser elongations: 4.8 cm and 5 cm (analytical results of 3.8 cm and 5.3 cm) under the same 20 kPa pressure level. The largest difference of 2 cm between the analytical and experimental results was observed for the 1 mm actuator with an inner radius of 5 mm, pressurized to 20 kPa. In this investigation, 20 kPa was chosen as the maximum allowable input pressure for all the bellow actuator samples. Further increasing the air pressure significantly contributed to complete extension of the actuator, rendering any results obtained thereafter irrelevant due to the loss of unfolding function. Moreover, excessive input pressure mainly caused irregular bulging effects.

Our experimental results highlight that the stability of the actuator during elongation is dependent on the dimensional aspects of the actuator design. Notably, a larger inner radius of the bellow roots results in shorter achievable elongation displacement under pressurization, accompanied by a more pronounced bulging effect at the same input pressure level. The estimation of elongation displacement exhibits a nonlinear trend during the initial stages of stretching the elastomer structure to unfold the bellow roots. As both input pressures and elongation displacement increase further, the estimation tends to approach a more linear relationship. As the input pressure gradually increases, the computational time required to obtain a solution in FEM simulations also increases. It was observed that convergence became challenging for all the actuator samples once the input pressure exceeded 20 kPa. This suggests that the actuator was nearing full unfolding, and additional computational time was necessary to investigate the effects of bellow wall membrane expansion when pressurized fluid was injected. It became evident that the analysis extended beyond the mere unfolding of the bellow roots.

## 5. Conclusions and Future Work

This article presents a bellow-based soft actuator that enables the active control of extension while maintaining a compact size. We equipped actuators with rigid ring constraints, and we developed an analytical model to quantify their elongation performance when subjected to pressurization. The experimental results, including elongation displacement measurements, are validated through finite element method (FEM) simulations and experimental testing in free space under fluidic pressurization. This comprehensive study facilitates the determination of optimal geometrical parameters for bellow-based actuators, enabling effective design optimization and performance prediction.

We acknowledge that our current models do not fully consider the dynamic behaviors of the elastomeric material as they do not account for the varying flow rates of input pressure into the undulated cavity. When subjected to normal cyclic loading, elastomer exhibits hysteresis, resulting in energy loss during deformation [32,33,34]. Previous studies have reported that at a slow pressure rate of 0.2 Hz, dynamic oscillations and hysteresis inside the elastomer can be avoided [35]. While our current models may suffice for certain robotic applications that operate at slow actuation speeds, such as around 0.2 Hz, they may not accurately capture the dynamic behavior of elastomer actuators under varying pressure rates. It is important to note that the limitations of our static models become more pronounced when actuation speed becomes a crucial factor. However, in specific applications like soft rehabilitation devices, where ROM takes precedence over speed, the current models can still be considered adequate [36,37]. Consequently, there may be variations in the experimental results when higher flow rates and the damping effects of the silicone rubber are considered. Therefore, relying solely on the developed quasi-static models for the dynamic control of soft actuators may result in inaccuracies, especially in scenarios where fast response times (e.g., less than 1 s) are required. These models primarily provide new insights into the mathematical relationship between input pressure and the elongation of bellow-based soft actuators in an analytical manner, addressing the challenge posed by the intricate undulated structure of the bellows.

To address this limitation, there is a need for advanced research and development in the dynamics modeling of soft actuators, specifically focusing on manipulators that require fast input and output responses. Accurate models, including techniques like efficient model order reduction and inverse dynamics modeling, hold potential and should be further investigated and expanded upon to enhance their applicability and effectiveness [38]. Eventually, in the realm of soft actuators, the utilization of dynamic closed-loop control can be advantageous for achieving robust responses to dynamic environments and external disturbances. This approach is better than data-driven and machine learning (ML) technologies in real-world scenarios [38]. Implementing dynamic models within closed-loop control follows a straightforward methodology, involving steps such as signal filtering of recorded data (e.g., bending angles, elongation displacement), dead zone implementation in the resting position to mitigate undesirable valve switching, and the incorporation of a feedback loop to the developed models. This feedback loop relates input pressure to the error between the desired actuator positions and the measured ongoing positions [35].

Therefore, our future plans involve incorporating the damping effects of silicone rubber into dynamic models, which will also account for the input pressure flow rate and its impact on elongation displacement. These dynamic modeling approaches will be integrated into the controller for bellow actuators, ensuring accurate and rapid response to input commands. The applications of such designs hold immense potential for advancing the field of soft robotic actuator designs and control, particularly for bio-inspired robots that require precise control of both actuator contraction and elongation, such as earthworm-inspired robots [25]. Furthermore, we also aim to design a soft robotic end-effector utilizing the bellow-based actuator design and investigate the force interaction between the actuator and the objects it manipulates. Our focus will be on conducting comprehensive analyses to model and quantify the output force of the actuators, contributing to the analytical design of bellow-based soft robotic manipulators. The design of bellow-based manipulators will also be optimized, particularly the tapered edges for 3D printing. This optimization process will enable us to leverage the benefits of 3D printing technology in the fabrication of our bellow-based soft robotic manipulator [39,40,41].

## Data Availability

Data are contained within the article.
